# An unusual thionyl chloride-promoted C−C bond formation to obtain 4,4'-bipyrazolones

**DOI:** 10.3762/bjoc.14.110

**Published:** 2018-06-04

**Authors:** Gernot A Eller, Gytė Vilkauskaitė, Algirdas Šačkus, Vytas Martynaitis, Ashenafi Damtew Mamuye, Vittorio Pace, Wolfgang Holzer

**Affiliations:** 1 Department of Pharmaceutical Chemistry, Faculty of Life Sciences, University of Vienna, Althanstrasse 14, A-1090 Vienna, Austria; 2 Department of Organic Chemistry, Kaunas University of Technology, Radvilėnų pl. 19, LT-50254 Kaunas, Lithuania; 3 Institute of Synthetic Chemistry, Kaunas University of Technology, K. Baršausko g. 59, LT-51423, Kaunas, Lithuania

**Keywords:** dimerization, NMR (^1^H, ^13^C, ^15^N), pyrazolones, thionyl chloride, X-ray structure analysis

## Abstract

Dialkyl 5,5'-dioxo-4,4'-bipyrazole-4,4'-dicarboxylates are readily obtained by the reaction of 5-hydroxypyrazole-4-carboxylates in refluxing thionyl chloride. The obtained diesters can be transformed into the corresponding 4,4'-bipyrazoles via alkaline hydrolysis and subsequent decarboxylation. Detailed NMR spectroscopic investigations (^1^H, ^13^C, ^15^N) were undertaken with all products prepared. Moreover, the structure of a representative 5,5'-dioxo-4,4'-bipyrazole-4,4'-dicarboxylate was confirmed by X-ray crystal structure analysis.

## Introduction

**Scheme 1:**
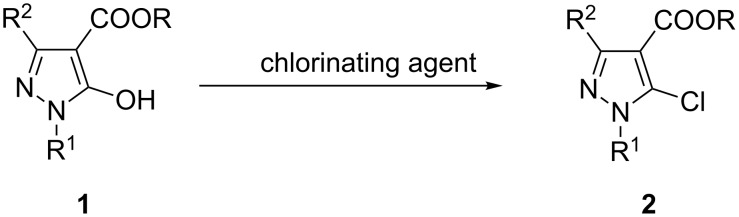
Envisaged approach for the synthesis of 5-chloropyrazole-4-carboxylates **2**.

Many biologically active substances, therein several drug molecules, agrochemicals, dyestuffs, compounds for optoelectronic purposes, complexing ligands and more contain a pyrazole nucleus [1–8]. Condensed pyrazoles are of special interest, as a commonly used example the phosphodiesterase 5 (PDE5) inhibitor sildenafil (Viagra^®^) can be mentioned [1]. In a series of former publications we described the synthesis of condensed pyrazole systems using various 4,5-disubstituted pyrazole derivatives as precursors for the annellation reaction [9–16]. Amongst these precursors 5-chloropyrazoles carrying C-substituents at the pyrazole C4 position, like 5-chloropyrazole-4-carbaldehydes or 4-esters, turned out to be particularly useful due to the easy conversion of the chloro substituent into other functional groups or its nature as a good leaving group in ring-closure reactions. In this respect we were interested in a convenient access to 1-substituted or 1,3-disubstituted 5-chloropyrazole-4-carboxylates required as valuable precursors for further functionalizations. Such compounds have been mainly prepared from the corresponding 5-aminopyrazole-4-carboxylates via (non-aqueous) diazotation and subsequent reaction with appropriate chlorine sources [17–18]. Additionally, some years ago we have presented a synthetic approach upon Vilsmeier reaction of 1-phenylpyrazolones with DMF/excessive POCl_3_ to afford 5-chloropyrazole-4-carbaldehydes, which were oxidized to the corresponding acids (KMnO_4_) and subsequently converted into the ethyl esters by treatment with EtOH/H_2_SO_4_ [15]. However, as the latter approach is tedious and the former one uses toxic substances we envisaged to convert the easily available 5-hydroxypyrazole-4-carboxylates **1** into the corresponding 5-chloro derivatives **2** by the action of an appropriate chlorinating agent such as POCl_3_ or SOCl_2_ (Scheme 1). Such conversions of a hydroxy (oxo) into a chloro function is very common with many N-heterocyclic systems, such as, for instance the transformations of 2-pyridones into 2-chloropyridines or 3-pyridazinones into 3-chloropyridazines [19–20]. In the course of the preparation of substituted 6*H*-pyrazolo[4,3-*d*][1,2]oxazoles we thus obtained the required 4-benzoyl-5-chloropyrazoles by treatment of the relevant 4-benzoyl-5-hydroxypyrazoles with POCl_3_ [21].

## Results and Discussion

### Chemistry

**Scheme 2:**
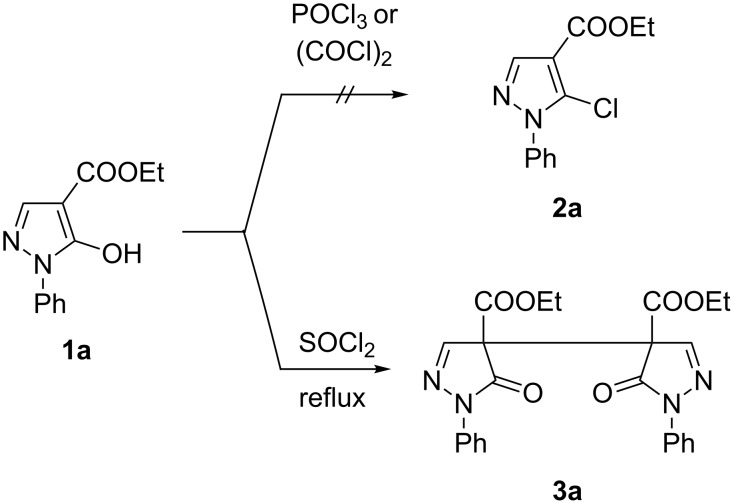
Synthesis of bipyrazolone **3a**.

**Figure 1:**
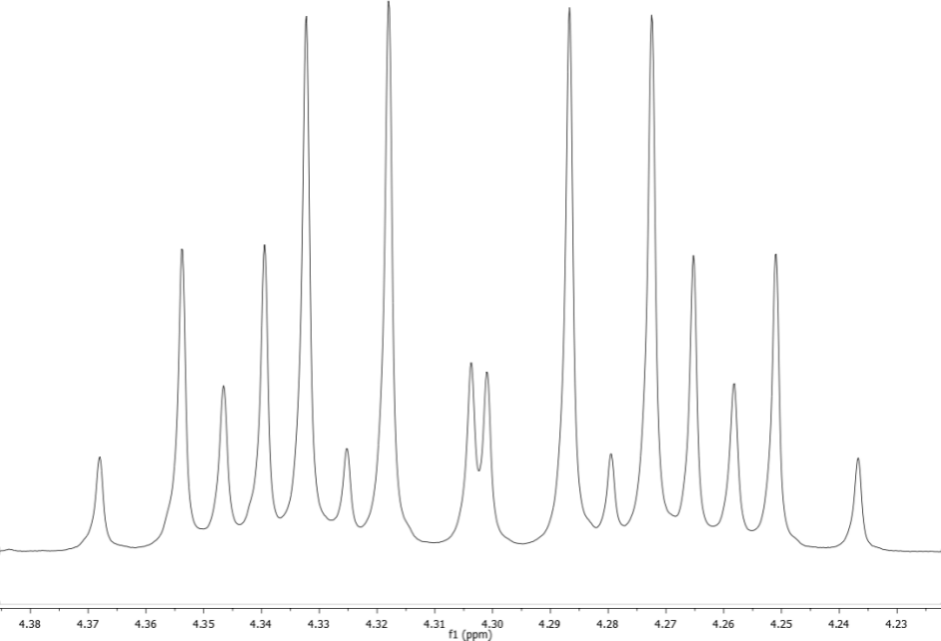
Signal of the OCH_2_ ester protons of reaction product **3a** (500 MHz, CDCl_3_).

However, the attempted reaction of ester **1a** (R^1^ = Ph, R^2^ = H, R = Et) with POCl_3_ left the starting material untouched, similarly by treatment of **1a** with oxalyl chloride no conversion occurred (Scheme 2). In contrast, treatment of **1a** with excessive thionyl chloride at reflux temperature resulted in a defined reaction product which, however, could not be the desired 5-chloro derivative **2a** according to – amongst others – a much too large chemical shift of pyrazole C5 (δ 165.6 ppm) compared to the expected one (δ 131.3 ppm) [15]. Moreover, the OCH_2_ protons revealed to be of diastereotopic character which hints to the presence of a chiral center in the molecule (Figure 1), while the molecular weight obtained by HRMS measurement ([M + Na]^+^ 485.1432) testified about the possible formation of a dimeric structure.

**Figure 2:**
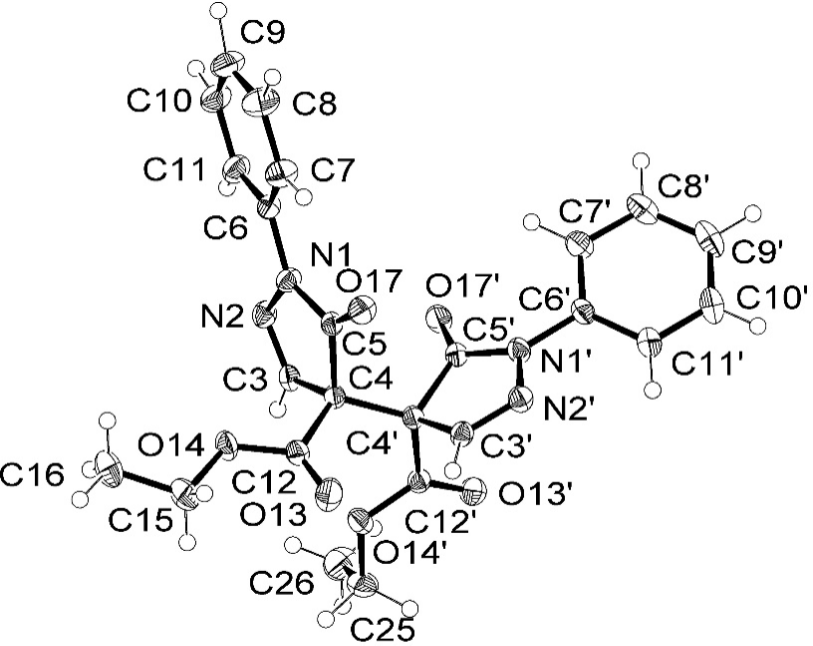
ORTEP-plot of the crystal structure of compound **3a** drawn with 50% displacement ellipsoids [(4*S*,4'*S*)-**3a** enantiomer is shown only]. The length of the C4–C4' bond connecting the two pyrazolone units is 1.544(3) Å. For details see the Supporting Information File 1.

Lastly, by X-ray crystal structure analysis the obtained product could be determined as the dimeric structure **3a** (Scheme 2, Figure 2). In addition, HRMS and elemental analysis confirmed the molecular formula. The non-equivalence of the OCH_2_ protons of the ester functions can be smoothly explained by the presence of an asymmetric carbon atom at pyrazole C4/C4'. As the NMR spectra displayed a single set of signals, regarding the stereochemistry a racemic mixture or the *meso*-form came into consideration.

**Figure 3:**
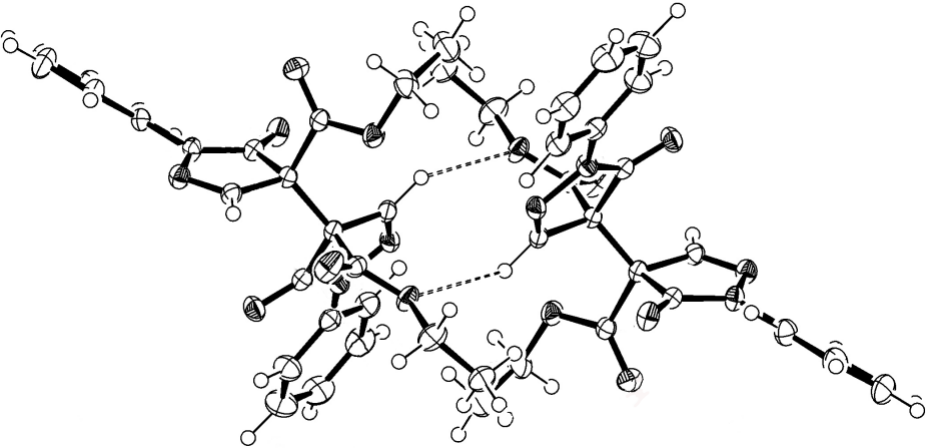
Arrangement (4*R*,4'*R)-* and (4*S*,4'*S*)-**3a** enantiomers in the crystal unit drawn with 50% displacement ellipsoids. The hydrogen bonds are shown by dashed lines. Supplementary crystallographic data for compound **3a** can be obtained free of charge from The Cambridge Crystallographic Data Centre via http://www.ccdc.cam.ac.uk/data_request/cif (CCDC 979625).

The single crystal X-ray analysis disclosed that the molecule of the newly obtained compound **3a** consists of two pyrazolone residues, which are directly connected to each other by a single covalent carbon–carbon bond between the asymmetric sp^3^-hybridized C4 and C4' carbon atoms to form a species with relative (4R*,4'R*)*-*configuration (Figure 2). The bond length of the single C4–C4' bond is 1.544(3) Å, while the dihedral angle C5–C4–C4'–C5' is 47.94°. The packaging of the chiral molecules (4*R*,4'*R*)**-3a** and (4*S*,4'*S*)**-3a** into a racemic crystal occurs in such a way that mirror enantiomers are interconnected to each other by weak intermolecular hydrogen bonds (C–H···O 2.523 Å, 130.64°, Figure 3).

**Scheme 3:**
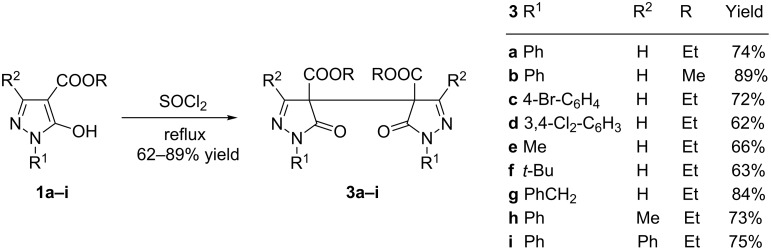
Synthesis of compounds **3a–i**.

In the following, related 5-hydroxypyrazol-4-carboxylates **1b–i** were subjected to the same reaction conditions (refluxing SOCl_2_) and in all cases the corresponding dimers of type **3** were obtained in moderate to good yields (Scheme 3).

**Scheme 4:**
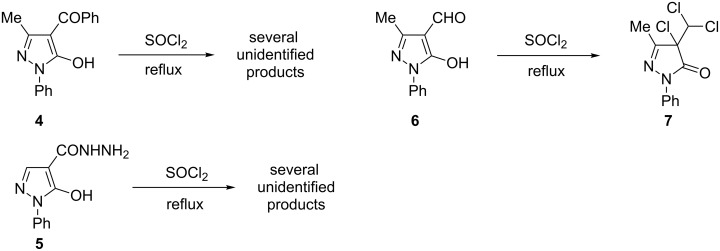
Reaction of different 5-hydroxypyrazoles with thionyl chloride.

In order to check if these dimerization reactions also occur with other 5-hydroxypyrazoles carrying a C=O function at pyrazole C4 we subjected ketone **4** and hydrazide **5** to the same reaction conditions. In both cases, a plethora of unidentified products resulted (Scheme 4). In contrast, with aldehyde **6** a reaction product could be isolated in moderate yield, which can be assigned to structure **7** considering NMR data and mass spectra (Scheme 4).

The dehydrogenative homocoupling of 2-pyrazolin-5-ones (or pyrazolidin-5-ones) is well documented in the literature and proceeds under different reaction conditions such as, for instance, by air oxidation [22], under O_2_ atmosphere using an O_2_ balloon [23], by organic peroxides [24], phenoxy radicals [25], by treatment with phenylhydrazine at high temperatures [26–27], by nitrosation and subsequent heating [28], by heating with an aqueous NaHSO_3_ solution [29], and by photochemistry [30–31]. In nearly all cases these reactions have been carried out with 2-pyrazolin-5-ones unsubstituted at pyrazole C4 position or with derivatives carrying an alkyl or aryl substituent at the latter carbon atom. In contrast, only very few examples are described for pyrazolones with a C=O substructure attached to pyrazole C4. Thus, 3-methyl-1-phenyl-4-toluoyl-5-pyrazolone (= (5-hydroxy-3-methyl-1-phenyl-1*H*-pyrazol-4-yl)(4-methylphenyl)methanone) upon treatment with oxovanadium(V) compounds afforded the corresponding 2,2',4,4'-tetrahydro-3*H*,3'*H*-4,4'-bipyrazole-3,3'-dione, whereas it was shown by EPR spectroscopy and by cyclic voltammetry that the reaction obviously proceeds via a radical mechanism [32]. To the best of our knowledge, the only such dimeric species with ester functions at pyrazole C4, namely diethyl 1,1'-dimethyl-5,5'-dioxo-1,1',5,5'-tetrahydro-4*H*,4'*H*-4,4'-bipyrazole-4,4'-dicarboxylate (structure **3** with R^1^ = Me, R^2^ = H, R = Et) has been obtained – amongst other reaction products – by UV–vis irradiation of 4-ethoxy-2-methyl-5-morpholino-3(2*H*)-pyridazinone (emorfazone) in acetonitrile [31].

**Scheme 5:**
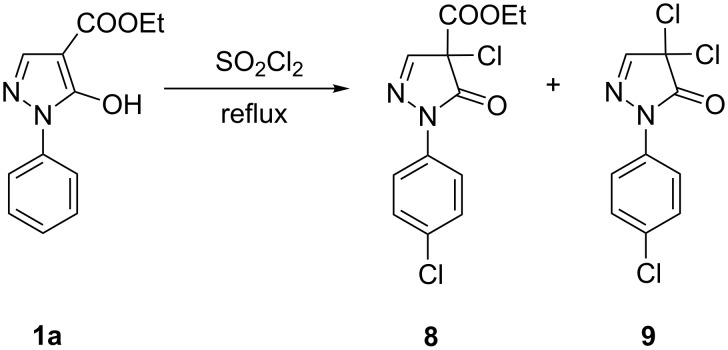
Reaction of **1a** with sulfuryl chloride.

Moreover, we investigated the reaction of **1a** with SO_2_Cl_2_. Here, two reaction products – **8** and **9** – were isolated, whereas in both cases chlorination not only at the pyrazole C4 but also in the 4-position of the phenyl ring took place (Scheme 5).

**Scheme 6:**
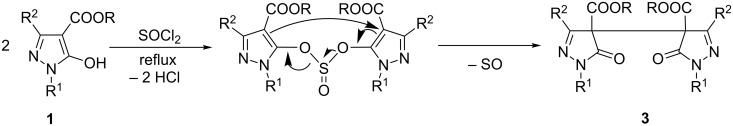
Possible reaction mechanism for the transformation **1** → **3**.

The reaction mechanism for the transformation **1** → **3** is unclear. Dimerization by air oxidation can be ruled out as performing the reaction under N_2_ atmosphere provided the same result. It should be mentioned that for the oxovanadium(V)-mediated dimerization of 4-aroyl-5-hydroxypyrazoles mentioned above a radical mechanism was postulated [32]. However, in Scheme 6 we propose a hypothetical mechanism comprising a redox cyclization of an intermediate di(pyrazolyl) sulfite under elimination of sulfur monoxide.

**Scheme 7:**
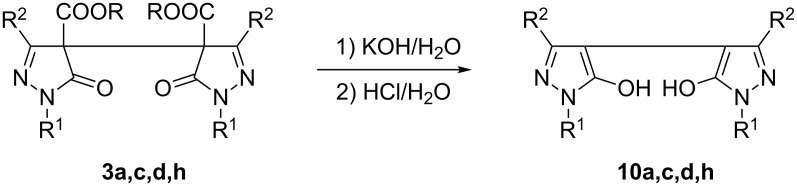
Preparation of bipyrazoles **10**.

Finally, it was shown by means of some selected examples, that compounds of type **3** can be converted into the corresponding bipyrazoles **10** upon alkaline hydrolysis and subsequent decarboxylation (Scheme 7). According to the NMR spectra, compounds **10** are obviously present as 5-hydroxypyrazoles due to the absence of a proton attached to pyrazole C4.

### NMR spectroscopic investigation

**Figure 4:**
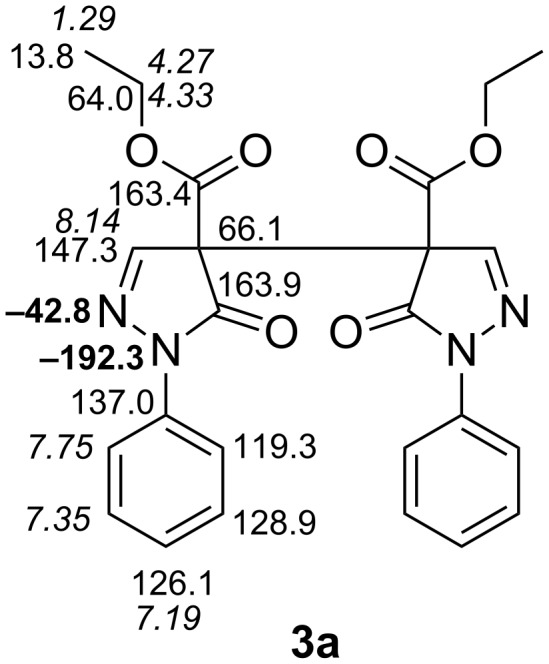
^1^H NMR (italics), ^13^C NMR (normal letters) and ^15^N NMR (in bold) chemical shifts of **3a** (in CDCl_3_).

In Supporting Information File 1 the NMR spectroscopic data of all compounds treated within this study are indicated. Full and unambiguous assignment of ^1^H, ^13^C and nearly all ^15^N NMR resonances was achieved by combining standard NMR techniques [33], such as fully ^1^H-coupled ^13^C NMR spectra, APT, gs-HSQC, gs-HMBC, gs-HSQC-TOCSY, COSY, TOCSY and NOESY spectroscopy. Figure 4 shows the thus assigned ^1^H, ^13^C and ^15^N NMR chemical shifts for model compound **3a**.

## Conclusion

The reaction of 5-hydroxypyrazole-4-carboxylates **1** with thionyl chloride does not lead to the corresponding 5-chloropyrazole congeners but induces dimerization to afford the relevant dialkyl 5,5'-dioxo-4,4'-bipyrazole-4,4'-dicarboxylates of type **3**.

## Supporting Information

File 1 Experimental details and compound characterization.
