# Supplementary material for: An unusual thionyl chloride-promoted C−C bond formation to obtain 4,4'-bipyrazolones
Source: Beilstein J Org Chem. 2018 Jun 4;14:1287–92. doi: 10.3762/bjoc.14.110 (PMC6009192; doi:10.3762/bjoc.14.110)
Supplement: File 1 — Experimental details and compound characterization. [file Beilstein_J_Org_Chem-14-1287-s001.pdf]

Supporting Information

for

An unusual thionyl chloride-promoted C−C bond formation to obtain 4,4'-bipyrazolones

Gernot A. Eller[1], Gytė Vilkauskaitė[1,2], Algirdas Šačkus[2,3], Vytas Martynaitis[2], Ashenafi Damtew Mamuye[1], Vittorio Pace[1] and Wolfgang Holzer*[1]

Address: [1]Department of Pharmaceutical Chemistry, Faculty of Life Sciences, University of Vienna, Althanstrasse 14, A-1090 Vienna, Austria, [2]Department of Organic Chemistry, Kaunas University of Technology, Radvilėnų pl. 19, LT-50254 Kaunas, Lithuania and [3]Institute of Synthetic Chemistry, Kaunas University of Technology, K. Baršausko g. 59, LT-51423, Kaunas, Lithuania

Email: Wolfgang Holzer* - wolfgang.holzer@univie.ac.at

* Corresponding author

Experimental details and compound characterization

1. General

Melting points were determined on a Reichert–Kofler hot-stage microscope and are uncorrected. Mass spectra were obtained on a Shimadzu QP 1000 instrument (EI, 70 eV) and on a Bruker maXis 4G instrument (ESI-TOF, HRMS). IR spectra (KBr pellets) were recorded on a PerkinElmer FTIR spectrum 1000 spectrophotometer and are reported in wave numbers (cm^{-1}). ^{1}H, ^{13}C and ^{15}N NMR spectra were recorded with a Bruker Avance III 400 spectrometer (400 MHz for ^{1}H, 100 MHz for ^{13}C, 40 MHz for ^{15}N), a Bruker Avance 500 spectrometer (500 MHz for ^{1}H, 125 MHz for ^{13}C, 50 MHz for ^{15}N) and a Varian UnityPlus 300 spectrometer (300 MHz for ^{1}H, 75 MHz for ^{13}C) at 297 K using "directly" detecting broadband observe (BBFO) probes. The center of the solvent signal was used as an internal standard which was related to TMS with δ 7.26 ppm (^{1}H in $CDCl_3$), δ 2.49 ppm (^{1}H in DMSO-d_6), δ 77.0 ppm (^{13}C in $CDCl_3$) and δ 39.5 ppm (^{13}C in DMSO-d_6). ^{15}N NMR spectra (gs-HMBC) were referenced against neat, external nitromethane. Digital resolutions were 0.2 Hz/data point in the ^{1}H NMR spectra and 0.3 Hz/data point in the ^{13}C NMR spectra. For column chromatographic separations Merck Kieselgel 60 (70–230 mesh) was used. Light

petroleum refers to the fraction with boiling point 40–65 °C. Elemental analyses were performed at the Microanalytical Laboratory, University of Vienna. Yields are not optimized.

General procedure for the preparation of 5-hydroxypyrazole-4-carboxylates 1a–f

K_2CO_3 (1.38 g, 10 mmol; when the corresponding hydrazine hydrochloride was employed 2.76 g, 20 mmol K_2CO_3 were applied) was dissolved in 75 mL of H_2O and then the appropriate hydrazine (hydrochloride) (10 mmol) was added. Diethyl (ethoxymethylene)malonate (or dimethyl (methoxymethylene)malonate in the synthesis of **1b**) (10 mmol) was added dropwise to the reaction mixture and the whole was refluxed for 2 h. Then the mixture was cooled to room temperature and extracted with 3 × 25 mL of EtOAc. For further procedure see below.

Ethyl 5-hydroxy-1-phenyl-1*H*-pyrazole-4-carboxylate (**1a**)

The aqueous phase was acidified with 6 N HCl to pH ≈ 2, the formed precipitate was filtered off, washed with water and recrystallized from aqueous EtOH. Yield: 1.97 g (85%), colorless crystals; mp 113 °C (aq EtOH) (lit. [1] mp: 118 °C). ^{1}H NMR (500 MHz, $CDCl_3$): δ (ppm) 1.39 (t, ^3J = 7.1 Hz, 3H, CH_3), 4.37 (q, ^3J = 7.1 Hz, 2H, CH_2), 7.32 (m, 1H, Ph H-4), 7.46 (m, 2H, Ph H-3,5), 7.77 (s, 1H, H-3), 7.80 (m, 2H, Ph H -2,6), 9.40 (br s, 1H, OH). ^{13}C NMR (125 MHz, $CDCl_3$): δ (ppm) 14.3 (CH_3), 60.7 (CH_2), 95.1 (C-4), 121.3 (Ph C-2,6), 127.1 (Ph C-4), 129.1 (Ph C-3,5), 137.5 (Ph C-1), 138.4 (C-3), 156.7 (C-5), 166.4 (CO). ^{15}N NMR (50 MHz, $CDCl_3$): δ (ppm) −185.1 (N-1), −100.8 (N-2).

Methyl 5-hydroxy-1-phenyl-1*H*-pyrazole-4-carboxylate (**1b**) [2]

The aqueous phase was acidified with 6 N HCl to pH ≈ 2, the formed precipitate was filtered off, washed with water and recrystallized from aqueous MeOH. Yield: 1.86 g (85%), brownish crystals; mp 158–159 °C (aq MeOH).^{1}H NMR (400 MHz, $CDCl_3$): δ (ppm) 3.90 (s, 3H, Me), 7.33 (m, 1H, Ph H-4), 7.47 (m, 2H, Ph H-3,5), 7.77 (s, 1H, H-3), 7.80 (m, 2H, Ph H-2,6), 9.70 (br s, 1H, OH). ^{13}C NMR (100 MHz, $CDCl_3$): δ (ppm) 51.6 (CH_3, ^1J = 147.6 Hz), 94.9 (C-4, $^2J_{C4,H3}$ = 9.7 Hz), 121.3 (Ph C-2,6), 127.1 (Ph C-4), 129.1 (Ph C-3,5), 137.5 (Ph C-1), 138.4 (C-3, ^1J = 191.6 Hz), 156.6 (C-5, $^3J_{C5,H3}$ = 4.4 Hz), 166.7 (CO, $^3J_{CO,Me}$ = 4.1 Hz). ^{15}N NMR (40 MHz, $CDCl_3$): δ (ppm) −184.9 (N-1), −100.5 (N-2). MS: *m/z* (%) = 218 $[M]^+$ (24), 186 (100), 118 (22), 91 (60), 77 (45). HRMS (ESI): calcd. for $C_{11}H_{11}N_2O_3^+$ $[M+H]^+$ 219.0764; found 219.0765.

Ethyl 1-(4-bromophenyl)-5-hydroxy-1*H*-pyrazole-4-carboxylate (**1c**)

From reaction of diethyl (ethoxymethene)malonate (2.16 g, 10 mmol), (4-bromophenyl)-hydrazine hydrochloride (2.23 g, 10 mmol) and K_2CO_3 (2.76 g, 20 mmol). After acidification with 6N HCl to pH ≈ 2 the mixture was extracted with 3 × 20 mL of EtOAc, the combined organic phases were washed with brine, dried over anhydrous Na_2SO_4, filtered and evaporated under reduced pressure. The residue was recrystallized from aqueous MeOH. Yield: 2.28 g (73%), yellowish crystals, mp 152–153 °C (aq MeOH) (lit. [3] 153–154 °C). ^{1}H NMR (400 MHz, $CDCl_3$): δ (ppm) 1.39 (t, ^3J = 7.1 Hz, 3H, CH_3), 4.37 (q, ^3J = 7.1 Hz, 2H, CH_2), 7.58

(m, 2H, Ph H-3,5), 7.71 (m, 2H, Ph H-2,6), 7.76 (s, 1H, H-3), 9.88 (br s, 1H, OH). ^{13}C NMR (100 MHz, $CDCl_3$): δ (ppm) 14.3 (CH_3, ^1J = 127.3 Hz, ^2J = 2.6 Hz), 60.9 (CH_2, ^1J = 148.1 Hz, ^2J = 4.5 Hz), 95.3 (C-4, $^2J_{C4,H3}$ = 9.5 Hz), 120.5 (Ph C-4), 122.5 (Ph C-2,6), 132.2 (Ph C-3,5), 136.6 (Ph C-1), 138.6 (C-3, ^1J = 191.8 Hz), 156.8 (C-5, $^3J_{C5,H3}$ = 4.5 Hz), 166.3 (CO). ^{15}N NMR (40 MHz, $CDCl_3$): δ (ppm) −186.9 (N-1), −101.5 (N-2). MS: *m/z* (%) = 310/312 $[M]^+$ (8/9), 264/266 (98/100), 169/171 (26/24), 155/157 (20/19). HRMS (ESI): calcd. for $C_{12}H_{11}BrN_2O_3Na^+$ $[M+Na]^+$ 332.9845; found 332.9847.

Ethyl 1-(3,4-dichlorophenyl)-5-hydroxy-1*H*-pyrazole-4-carboxylate (**1d**).

From reaction of diethyl (ethoxymethylene)malonate (2.16 g, 10 mmol), (3,4-dichlorophenyl)hydrazine hydrochloride (2.13 g, 10 mmol) and K_2CO_3 (2.76 g, 20 mmol). After acidification with 6N HCl to pH ≈ 2 the formed precipitate was filtered off, washed with water and recrystallized from aqueous EtOH. Yield: 2.29 g (76%), yellow crystals, mp 152–154 °C (aq EtOH). ^{1}H NMR (400 MHz, $CDCl_3$): δ (ppm) 1.39 (t, ^3J = 7.1 Hz, 3H, CH_3), 4.38 (q, ^3J = 7.1 Hz, 2H, CH_2), 7.52 (dd, ^3J = 8.8 Hz, ^5J = 0.3 Hz, 1H, Ph H-5), 7.73 (dd, ^3J = 8.8 Hz, ^4J = 2.5 Hz, 1H, Ph H-6), 7.76 (s, 1H, H-3), 8.01 (dd, ^4J = 2.5 Hz, ^5J = 0.3 Hz, 1H, Ph H-2), 9.80 (br s, 1H, OH). ^{13}C NMR (100 MHz, $CDCl_3$): δ (ppm) 14.3 (CH_3, ^1J = 127.3 Hz, $^2J_{CH3,CH2}$ = 2.6 Hz), 61.0 (CH_2, ^1J = 148.2 Hz, $^2J_{CH2,CH3}$ = 4.5 Hz), 95.5 (C-4, $^2J_{C4,H3}$ = 9.6 Hz), 119.8 (Ph-C6), 122.6 (Ph-C2), 130.7 (Ph C-5, ^1J = 169.4 Hz), 130.7 (Ph C-4), 133.2 (Ph-C3), 136.9 (Ph-C1), 138.9 (C-3, ^1J = 192.1 Hz), 157.0 (C-5, $^3J_{C5,H3}$ = 4.6 Hz), 166.2 (CO, $^3J_{CO,CH2}$ = 3.2 Hz). ^{15}N NMR (50 MHz, $CDCl_3$): δ (ppm) −188.3 (N-1), −102.0 (N-2). IR (KBr): 3415 (OH), 1711 (C=O) cm^{-1}. MS: *m/z* (%) = 300/302/304 $[M]^+$ (9/6/1), 254/256/258 (100/78/9). HRMS (ESI): calcd. for $C_{12}H_{10}Cl_2N_2O_3Na^+$ $[M+Na]^+$ 322.9961; found 322.9962. Anal. Calcd for $C_{12}H_{10}Cl_2N_2O_3$: C, 47.86; H, 3.35; N, 9.30, found: C, 47.96; H, 3.31; N, 9.28.

Ethyl 5-hydroxy-1-methyl-1*H*-pyrazole-4-carboxylate (**1e**)

Methylhydrazine (1.38 g, 30 mmol) was added to diethyl (ethoxymethylene)malonate (6.49 g, 30 mmol) in ethanol (50 mL) at 0 °C and the mixture was refluxed overnight. The solvent was removed under reduced pressure, the residue was washed with light petroleum and recrystallized from aqueous MeOH. Yield: 3.25 g (64%), colorless crystals, mp 134–135 °C (aq MeOH) (lit. [4] 138–139 °C). ^{1}H NMR (400 MHz, $CDCl_3$): δ (ppm) 1.33 (t, ^3J = 7.1 Hz, 3H, CH_3), 3.64 (s, 3H, 1-Me), 4.29 (q, ^3J = 7.1 Hz, 2H, CH_2), 7.56 (s, 1H, H-3), 7.98 (br s, 1H, OH). ^{13}C NMR (100 MHz, $CDCl_3$): δ (ppm) 14.3 (CH_3), 33.3 (1-Me), 60.3 (CH_2), 94.0 (C-4), 137.5 (C-3), 156.4 (C-5), 165.8 (CO). ^{15}N NMR (40 MHz, $CDCl_3$): δ (ppm) −202.4 (N-1), −100.0 (N-2).

Ethyl 1-*tert*-butyl-5-hydroxy-1*H*-pyrazole-4-carboxylate (**1f**)

From reaction of diethyl (ethoxymethylene)malonate (2.16 g, 10 mmol), *tert*-butylhydrazine hydrochloride (1.25 g, 10 mmol) and K_2CO_3 (2.76 g, 20 mmol). After acidification with 6 N HCl to pH ≈ 2 the mixture was extracted with 3 × 20 mL of EtOAc, the combined organic phases were washed with brine, dried over anhydrous Na_2SO_4, filtered and evaporated under reduced pressure. Yield: 0.96 g (45%), colorless oil. ^{1}H NMR (400 MHz, $CDCl_3$): δ (ppm) 1.34 (t, ^3J = 7.1 Hz, 3H, CH_3), 1.60 (s, 9H, CH_3), 4.31 (q, ^3J = 7.1 Hz, 2H, CH_2), 7.52 (s, 1H, H-3), 9.50 (br s, 1H, OH). ^{13}C NMR (100 MHz, $CDCl_3$): δ (ppm) 14.4 (CH_3, ^1J = 127.1 Hz, $^2J_{CH3,CH2}$ = 2.6 Hz), 28.5 ($C(\underline{C}H_3)_3$), 59.4 ($\underline{C}(CH_3)_3$), 60.3 (CH_2, ^1J = 147.8 Hz, $^2J_{CH2,CH3}$ = 4.5 Hz), 94.6 (C-4, $^2J_{C4,H3}$ = 9.7 Hz), 135.7 (C-3, ^1J = 190.3 Hz), 157.1 (C-5, $^3J_{C5,H3}$ = 4.3 Hz), 166.7 (CO). ^{15}N NMR (40 MHz, $CDCl_3$): δ (ppm) −174.3 (N-1), −102.3 (N-2). MS: *m/z* (%)

= 212 $[M]^+$ (22), 151 (29), 111 (40), 110 (100), 57 (49), 53 (29), 41 (31). HRMS (ESI): calcd. for $C_{10}H_{16}N_2O_3Na^+$ $[M+Na]^+$ 235.1053. Found: 235.1063.

Ethyl 1-benzyl-5-hydroxy-1*H*-pyrazole-4-carboxylate (**1g**)

The synthesis and spectroscopic data of **1g** have been reported by us previously [5].

Ethyl 5-hydroxy-3-methyl-1-phenyl-1*H*-pyrazole-4-carboxylate (**1h**).

To a solution of 3-methyl-1-phenyl-2-pyrazolin-5-one (13.94 g, 80 mmol) in dioxane (70 mL) $Ca(OH)_2$ (11.85 g, 160 mmol) was added and ethyl chloroformate (8.68 g, 80 mmol) was added dropwise. After refluxing for 45 min the mixture was cooled and 5% HCl (≈ 200 mL until pH ≈ 3) was added. The mixture was cooled and the precipitated crystals were filtered off. The dried crude product was recrystallized from EtOH/H_2O 4:1. Yield: 7.69 g (39%), pink crystals, mp 120–122 °C (aq EtOH) (lit.[6] 114–115 °C). ^{1}H NMR (500 MHz, DMSO-d_6): δ (ppm) 1.27 (t, ^3J = 7.1 Hz, 3H, CH_3), 2.31 (s, 3H, 3-Me), 4.21 (q, ^3J = 7.1 Hz, 2H, CH_2), 7.30 (m, 1H, Ph H-4), 7.46 (m, 2H, Ph H-3,5), 7.68 (m, 2H, Ph H-2,6), 11.20 (br s, 1H, OH). ^{13}C NMR (125 MHz, DMSO-d_6): δ (ppm) 14.4 (CH_3, ^1J = 126.7 Hz, $^2J_{CH3,CH2}$ = 2.6 Hz), 14.7 (3-Me, ^1J = 128.6 Hz), 59.1 (CH_2, ^1J = 147.3 Hz, $^2J_{CH2,CH3}$ = 4.5 Hz), 94.7 (C-4, $^3J_{C4,3\text{-}Me}$ = 2.5 Hz), 121.7 (Ph C-2,6), 126.4 (Ph C-4), 129.0 (Ph C-3,5), 137.6 (Ph C-1), 149.1 (C-3, $^2J_{C3,3\text{-}Me}$ = 6.8 Hz), 155.6 (C-5), 163.3 (CO, $^2J_{CO,CH2}$ = 3.1 Hz). ^{15}N NMR (50 MHz, DMSO-d_6): δ (ppm) −189.3 (N-1), N-2 was not found.

Ethyl 5-hydroxy-1,3-diphenyl-1*H*-pyrazole-4-carboxylate (**1i**)

Diethyl benzoylmalonate (2.64 g, 10 mmol) was dissolved in 10 mL of acetic acid at room temperature. A solution of phenylhydrazine (4.33 g, 40 mmol) in 10 mL of 70% acetic acid was added dropwise. The reaction mixture was stirred for 2 days, during this time a precipitate formed. The precipitate was filtered off, washed with water and air dried to afford the title compound which was used in the next reaction step without further purification. Yield: 2.76 g (90%), colorless crystals, mp 98–99 °C (lit.[7] 98 °C). ^{1}H NMR (400 MHz, $CDCl_3$): δ (ppm) 1.31 (t, ^3J = 7.1 Hz, 3H, CH_3), 4.35 (q, ^3J = 7.1 Hz, 2H, CH_2), 7.33 (m, 1H, NPh H-4), 7.42 (m, 3H, CPh H-3,4,5), 7.48 (m, 2H, NPh H-3,5), 7.82 (m, 2H, CPh H-2,6), 7.91 (m, 2H, NPh H-2,6), 10.80 (br s, 1H, OH). ^{13}C NMR (100 MHz, $CDCl_3$): δ (ppm) 14.0 (CH_3), 61.0 (CH_2), 92.5 (C-4), 121.4 (NPh C-2,6), 127.0 (NPh C-4), 127.7 (CPh C-3,5), 128.8 (CPh C-4), 129.1 (NPh C-3,5), 129.2 (CPh C-2,6), 132.5 (CPh C-1), 137.5 (NPh C-1), 150.4 (C-3), 158.4 (C-5), 167.2 (CO). ^{15}N NMR (40 MHz, $CDCl_3$): δ (ppm) −188.1 (N-1), −104.4 (N-2).

General procedure for the preparation of bipyrazoles 3

5-Hydroxypyrazole-4-ester **2** (10.0 mmol) and $SOCl_2$ (7.25 mL, 11.9 g, 100 mmol) were refluxed for 3 h. Then the excess $SOCl_2$ was removed under reduced pressure, toluene (15 mL) was added, and the solvent was removed under reduced pressure. The crude product was purified as outlined below.

Diethyl *rac*-(4*R*,4'*R*)-5,5'-dioxo-1,1'-diphenyl-1,1',5,5'-tetrahydro-4*H*,4'*H*-[4,4'-bipyrazole]-4,4'-dicarboxylate (**3a**).

The residual oil crystallized on standing. Recrystallization from cyclohexane/light petroleum

afforded 1.71 g (74%) of colorless crystals, mp 77–78 °C. ^{1}H NMR (500 MHz, $CDCl_3$): δ (ppm) 1.29 (t, ^3J = 7.1 Hz, 6H, CH_3), 4.27 (dq, ^2J = 10.7 Hz, ^3J = 7.1 Hz, 2H, OCH_2), 4.33 (dq, ^2J = 10.7 Hz, ^3J = 7.1 Hz, 2H, OCH_2), 7.19 (m, 2H, Ph H-4), 7.35 (m, 4H, Ph H-3,5), 7.75 (m, 4H, Ph H-2,6), 8.14 (s, 2H, pyrazole H-3). ^{13}C NMR (125 MHz, $CDCl_3$): δ (ppm) 13.8 (CH_3, ^1J = 127.8 Hz, $^2J_{CH3,CH2}$ = 2.6 Hz), 64.0 (CH_2, ^1J = 149.5 Hz, $^2J_{CH2,CH3}$ = 4.4 Hz), 66.1 (pyrazole C-4, $^2J_{C4,H3}$ = 9.6 Hz), 119.3 (Ph C-2,6), 126.1 (Ph C-4), 128.9 (Ph C-3,5), 137.0 (Ph C-1), 147.3 (pyrazole C-3, ^1J = 209.6 Hz), 163.4 (ester CO, $^3J_{CO,CH2}$ = 3.4 Hz), 163.9 (pyrazole C-5, $^3J_{C5,H3}$ = 3.0 Hz). ^{15}N NMR (50 MHz, $CDCl_3$): δ (ppm) −192.3 (N-1), −42.8 (N-2). IR (KBr): 1714 (C5=O), 1754 (ester CO) cm^{-1}. ESI-MS: m/z (%) = 463 $[M+H]^+$ (100). Anal. Calcd for $C_{24}H_{22}N_4O_6$: C, 62.33; H, 4.79; N, 12.12; found: C, 62.39; H, 4.89; N, 12.18. HRMS (ESI): calcd. for $C_{24}H_{22}N_4O_6Na^+$ $[M+Na]^+$ 485.1432; found 485.1431.

Dimethyl 5,5'-dioxo-1,1'-diphenyl-1,1',5,5'-tetrahydro-4*H*,4'*H*-[4,4'-bipyrazole]-4,4'-dicarboxylate (**3b**)

The remaining residue was purified by column chromatography (SiO_2, CH_2Cl_2/light petroleum 3:1, v/v). Yield: 1.93 g (89%), yellowish crystals, mp 132–134 °C. ^{1}H NMR (400 MHz, $CDCl_3$): δ (ppm) 3.85 (s, 6H, OCH_3), 7.19 (m, 2H, Ph-4), 7.35 (m, 4H, Ph H-3,5), 7.75 (m, 4H, Ph H-2,6), 8.13 (s, 2H, H-5). ^{13}C NMR (100 MHz, $CDCl_3$): δ (ppm) 54.5 (OCH_3, ^1J = 148.2 Hz), 65.9 (pyrazole C-4, $^2J_{C4,H3}$ = 9.7 Hz), 119.3 (Ph C-2,6), 126.1 (Ph C-4), 128.9 (Ph C-3,5), 137.0 (Ph C-1), 147.0 (pyrazole C-3, ^1J = 209.6 Hz), 164.0 (ester CO, $^3J_{CO,CH3}$ = 4.1 Hz), 163.7 (pyrazole C-5, $^3J_{C5,H3}$ = 2.9 Hz). ^{15}N NMR (40 MHz, $CDCl_3$): δ (ppm) −191.9 (N-1), −42.0 (N-2). ESI-MS: *m/z* (%) = 435 $[M+H]^+$ (100). HRMS (ESI): calcd. for $C_{22}H_{18}N_4O_6Na^+$ $[M+Na]^+$ 457.1119; found 457.1122.

Diethyl 1,1'-bis(4-bromophenyl)-5,5'-dioxo-1,1',5,5'-tetrahydro-4*H*,4'*H*-[4,4'-bipyrazole]-4,4'-dicarboxylate (**3c**)

The remaining residue was thoroughly washed with *n*-hexane and dried in vacuo. Yield: 2.24 g (72%), yellowish crystals, mp 44–45 °C. ^{1}H NMR (400 MHz, $CDCl_3$): δ (ppm) 1.28 (t, ^3J = 7.1 Hz, 6H, CH_3), 4.28 (dq, ^2J = 10.7 Hz, ^3J = 7.1 Hz, 2H, OCH_2), 4.35 (dq, ^2J = 10.7 Hz, ^3J = 7.1 Hz, 2H, OCH_2), 7.47 (m, 2H, Ph H-3,5), 7.66 (m, 4H, Ph H-2,6), 8.12 (s, 2H, H-3). ^{13}C NMR (100 MHz, $CDCl_3$): δ (ppm) 13.8 (CH_3, ^1J = 127.8 Hz, $^2J_{CH3,CH2}$ = 2.6 Hz), 64.2 (CH_2, ^1J = 149.7 Hz, $^2J_{CH2,CH3}$ = 4.4 Hz), 66.1 (pyrazole C-4, $^2J_{C4,H3}$ = 9.5 Hz), 119.2 (Ph C-4), 120.6 (Ph C-2,6), 132.0 (Ph C-3,5), 136.0 (Ph C-1), 147.6 (pyrazole C-3, ^1J = 209.9 Hz), 163.1 (ester CO, $^3J_{CO,CH2}$ = 3.4 Hz), 163.9 (C-5, $^3J_{C5,H3}$ = 2.9 Hz). ^{15}N NMR (40 MHz, $CDCl_3$): δ (ppm) −193.5 (N-1), −43.8 (N-2). ESI-MS: *m/z* (%) = 621 $[M+H]^+$ (33), 540 (29), 500 (430), 499 (92), 482 (31), 484 (100), 321 (95). HRMS (ESI): calcd. for $C_{24}H_{20}Br_2N_4O_6Na^+$ $[M+Na]^+$: 640.9642; found: 640.9630.

Diethyl 1,1'-bis(3,4-dichlorophenyl)-5,5'-dioxo-1,1',5,5'-tetrahydro-4*H*,4'*H*-[4,4'-bipyrazole]-4,4'-dicarboxylate (**3d**)

The remaining residue was purified by column chromatography (SiO_2, CH_2Cl_2/light petroleum 7:3, v/v. Yield: 1.86 g (62%), colorless crystals, mp 51–54 °C. ^{1}H NMR (300 MHz, $CDCl_3$): δ (ppm) 1.30 (t, ^3J = 7.1 Hz, 6H, CH_3), 4.28 (dq, ^2J = 10.8 Hz, ^3J = 7.1 Hz, 2H, OCH_2), 4.35 (dq, ^2J = 10.8 Hz, ^3J = 7.1 Hz, 2H, OCH_2), 7.41 (d, ^3J = 8.9 Hz, 2H, Ph H-5),

7.68 (dd, ^3J = 8.9 Hz, ^4J = 2.6 Hz, 2H, Ph H-6), 7.97 (d, ^4J = 2.6 Hz, 2H, Ph H-2), 8.14 (s, 2H, pyrazole H-3). ^{13}C NMR (75 MHz, $CDCl_3$): δ (ppm) 13.9 (CH_3, ^1J = 127.8 Hz, $^2J_{CH3,CH2}$ = 2.6 Hz), 64.4 (OCH_2, ^1J = 149.8 Hz, $^2J_{CH2,CH3}$ = 4.4 Hz), 66.2 (pyrazole C-4, $^2J_{C4,H3}$ = 9.6 Hz), 117.9 (Ph C-6), 120.6 (Ph C-2), 129.6 (Ph C-4), 130.6 (Ph C-5), 133.0 (Ph C-3), 136.2 (Ph C-1), 147.8 (pyrazole C-3, ^1J = 210.3 Hz), 162.8 (ester CO, $^3J_{CO,CH2}$ = 3.4 Hz), 163.9 (pyrazole C-5, $^3J_{C5,H3}$ = 3.0 Hz). ^{15}N NMR (50 MHz, $CDCl_3$): δ (ppm) −194.5 (N-1), −44.7 (N-2). IR (KBr): 1717 (C5=O), 1750 (ester CO) cm^{-1}. ESI-MS: *m/z* (%) = 601 $[M+H]^+$ (100), 598 (78). Anal. Calcd for $C_{24}H_{18}Cl_4N_4O_6$: C, 48.02; H, 3.02; N, 9.33; found: C, 48.11; H, 3.04; N, 9.25. HRMS (ESI): calcd. for $C_{24}H_{18}Cl_4N_4O_6Na^+$ $[M+Na]^+$:620.9873; found: 620.9868.

Diethyl 1,1'-dimethyl-5,5'-dioxo-1,1',5,5'-tetrahydro-4*H*,4'*H*-[4,4'-bipyrazole]-4,4'-dicarboxylate (**3e**).

The remaining residue was purified by column chromatography (SiO_2, EtOAc/light petroleum = 1:1, v/v. Yield: 1.124 g (66%) colorless crystals, mp 129–130 °C. ^{1}H NMR (400 MHz, $CDCl_3$): δ (ppm) 1.25 (t, ^3J = 7.1 Hz, 6H, CH_3), 3.27 (s, 6H, 1-Me), 4.21 (dq, ^2J = 10.8 Hz, ^3J = 7.1 Hz, 2H, OCH_2), 4.23 (dq, ^2J = 10.8 Hz, ^3J = 7.1 Hz, 2H, OCH_2), 7.83 (s, 2H, H-3). ^{13}C NMR (100 MHz, $CDCl_3$): δ (ppm) 13.8 (CH_3, ^1J = 127.7 Hz, $^2J_{CH3,CH2}$ = 2.7 Hz), 31.8 (1-Me, ^1J = 140.8 Hz), 63.6 (OCH_2, ^1J = 149.4 Hz, $^2J_{CH2,CH3}$ = 4.4 Hz), 64.4 (pyrazole C-4, $^2J_{C4,H3}$ = 9.7 Hz), 146.7 (pyrazole C-3, ^1J = 209.1 Hz), 163.6 (ester CO, $^3J_{CO,CH2}$ = 3.4 Hz), 165.8 (pyrazole C-5, $^3J_{C5,H3}$ = 2.8 Hz, $^3J_{C5,NCH3}$ = 2.3 Hz). ^{15}N NMR (40 MHz, $CDCl_3$): δ (ppm) −209.4 (N-1), −40.0 (N-2). ESI-MS: *m/z* (%) = 339 $[M+H]^+$ (100). HRMS (ESI): calcd. for $C_{14}H_{18}N_4O_6Na^+$ $[M+Na]^+$ 361.1119; found 361.1121.

Diethyl 1,1'-di-*tert*-butyl-5,5'-dioxo-1,1',5,5'-tetrahydro-4*H*,4'*H*-[4,4'-bipyrazole]-4,4'-dicarboxylate (**3f**)

The remaining residue was thoroughly washed with *n*-hexane. Yield: 1.33 g (63%) colorless crystals, mp 80–81 °C. ^{1}H NMR (400 MHz, $CDCl_3$): δ = 1.23 (t, ^3J = 7.1 Hz, 6H, ester CH_3), 1.44 (s, 18H, CH_3), 4.10–4.35 (m, 4H, CH_2), 7.71 (s, 2H, pyrazole H-3). ^{13}C NMR (100 MHz, $CDCl_3$): δ (ppm) 13.8 (ester CH_3, ^1J = 127.6 Hz, $^2J_{CH3,CH2}$ = 2.7 Hz), 27.8 (CH_3, ^1J = 127.6 Hz, $^3J_{CH3,CH3}$ = 3.4 Hz), 58.3 ($\underline{C}(CH_3)_3$), 63.1 (OCH_2, ^1J = 149.2 Hz, $^2J_{CH2,CH3}$ = 4.4 Hz), 65.8 (pyrazole C-4, $^2J_{C4,H3}$ = 9.7 Hz), 145.1 (pyrazole C-3, ^1J = 208.2 Hz), 164.0 (ester CO, $^3J_{CO,CH2}$ = 3.3 Hz), 165.9 (pyrazole C-5, $^3J_{C5,H3}$ = 2.8 Hz). ^{15}N NMR (40 MHz, $CDCl_3$): δ (ppm) −184.2 (N-1), −40.1 (N-2). MS: *m/z* (%) = 422 $[M]^+$ (9), 407 (18), 231 (16), 166 (33), 165 (30), 151 (30), 111 (37), 110 (65), 57 (100), 56 (21), 41 (40). HRMS (ESI): calcd. for $C_{20}H_{30}N_4O_6Na$ $[M+Na]^+$: 445.2058; found: 445.2063.

Diethyl 1,1'-dibenzyl-5,5'-dioxo-1,1',5,5'-tetrahydro-4*H*,4'*H*-[4,4'-bipyrazole]-4,4'-dicarboxylate (**3g**)

The remaining residue was recrystallized from aqueous EtOH. Yield: 2.06 g (84%) colorless crystals, mp 106–107 °C (aq EtOH). ^{1}H NMR (400 MHz, $CDCl_3$): δ (ppm) 1.18 (t, ^3J = 7.1 Hz, 6H, CH_3), 4.18 (dd, ^2J = 10.7 Hz, ^3J = 7.1 Hz, 2H, OCH_2), 4.21 (dd, ^2J = 10.7 Hz, ^3J = 7.1 Hz, 2H, OCH_2), 4.66 (A-part of an AB-system, $^2J_{AB}$ = 15.2 Hz, 2H, NCH_2), 4.80 (A-part of an AB-system, $^2J_{AB}$ = 15.2 Hz, 2H, NCH_2), 7.23 (m, 4H, Ph H-2,6), 7.29 (m, 6H, Ph H-3,4,5), 7.86 (s, 2H, pyrazole H-3). ^{13}C NMR (100 MHz, $CDCl_3$): δ (ppm) 13.7 (CH_3, ^1J = 127.7 Hz, $^2J_{CH3,CH2}$ = 2.6 Hz), 48.4 (NCH_2, ^1J = 140.8 Hz), 63.6 (OCH_2, ^1J = 149.4 Hz, $^2J_{CH2,CH3}$ = 4.4 Hz), 64.0 (pyrazole C-4, $^2J_{C4,H3}$ = 9.8 Hz), 127.77 (Ph C-2,6), 127.79 (Ph C-4), 128.5 (Ph C-3,5), 135.5 (Ph C-1), 146.7 (pyrazole C-3, ^1J = 209.2 Hz), 163.6 (ester CO, $^3J_{CO,CH2}$ = 3.3 Hz), 165.6 (pyrazole C-5, $^3J_{C5,H3}$ = 2.7 Hz, $^3J_{C3,NCH2}$ = 2.7 Hz). ^{15}N NMR (40 MHz, $CDCl_3$): δ

(ppm) −197.8 (N-1), −41.2 (N-2). MS: *m/z* (%) = 490 $[M]^+$ (0.21), 345 (27), 91 (100). Anal. Calcd for $C_{26}H_{26}N_4O_6$: C, 63.66; H, 5.34; N, 11.42; found: C, 63.41; H, 5.03; N, 11.39.

Diethyl 3,3'-dimethyl-5,5'-dioxo-1,1'-diphenyl-1,1',5,5'-tetrahydro-4*H*,4'*H*-[4,4'-bipyrazole]-4,4'-dicarboxylate (**3h**)

The residue was purified by column chromatography (SiO_2, EtOAc/light petroleum = 7:3, v/v. Yield: 1.79 g (73%), colorless crystals, mp 56–57 °C. ^1H NMR (300 MHz, $CDCl_3$): δ (ppm) 1.30 (t, ^3J = 7.1 Hz, 6H, CH_3), 2.55 (s, 6H, 3-Me), 4.25 (dq, ^2J = 10.7 Hz, ^3J = 7.1 Hz, 2H, OCH_2), 4.38 (dq, ^2J = 10.7 Hz, ^3J = 7.1 Hz, 2H, OCH_2), 7.15 (m, 2H, Ph H-4), 7.32 (m, 4H, Ph H-3,5), 7.73 (m, 4H, Ph H-2,6). ^{13}C NMR (75 MHz, $CDCl_3$): δ (ppm) 13.9 (CH_3, ^1J = 127.6 Hz, $^2J_{CH3,CH2}$ = 2.6 Hz), 19.4 (3-Me, ^1J = 130.8 Hz), 63.4 (OCH_2, ^1J = 149.3 Hz, $^2J_{CH2,CH3}$ = 4.4 Hz), 67.8 (pyrazole C-4, $^3J_{C4,Me}$ = 2.6 Hz), 119.4 (Ph C-2,6), 125.8 (Ph C-4), 128.7 (Ph C-3,5), 137.0 (Ph C-1), 157.5 (pyrazole C-3, $^2J_{C3,Me}$ = 8.0 Hz), 163.2 (ester CO, $^3J_{CO,CH2}$ = 3.5 Hz), 165.0 (pyrazole C-5). ^{15}N NMR (50 MHz, $CDCl_3$): δ (ppm) −195.3 (N-1), −57.8 (N-2). IR (KBr): 1716 (C5=O), 1753 (ester CO) cm^{-1}. MS: *m/z* (%) = 490 $[M]^+$ (7),245 (18), 199 (73), 91 (27), 77 (100). ESI-MS: *m/z* (%) = 491 $[M+H]^+$ (100). Anal. Calcd for $C_{26}H_{26}N_4O_6$: C, 63.66; H, 5.34; N, 11.42; found: C, 63.75; H, 5.28; N, 11.52. HRMS (ESI): calcd. for $C_{26}H_{27}N_4O_6$ $[M+H]^+$ 491.1925; found 491.1925.

Diethyl 5,5'-dioxo-1,1',3,3'-tetraphenyl-1,1',5,5'-tetrahydro-4*H*,4'*H*-[4,4'-bipyrazole]-4,4'-dicarboxylate (**3i**)

The remaining residue was thoroughly washed with *n*-hexane and dried in vacuo. Yield: 2.30 g (75%), colorless crystals, mp 66–68 °C. ^1H NMR (400 MHz, $CDCl_3$): δ (ppm) 1.28 (t, ^3J = 7.1 Hz, 6H, CH_3), 4.38 (q, ^3J = 7.1 Hz, 4H, OCH_2), 7.15 (m, 2H, NPh H-4), 7.16 (m, 2H, CPh H-4), 7.19 (m, 4H, CPh H-3,5), 7.27 (m, 4H, NPh H-3,5), 7.55 (m, 4H, NPh H-2,6), 7.67 (m, 4H, CPh H-2,6). ^{13}C NMR (100 MHz, $CDCl_3$): δ (ppm) 13.7 (CH_3), 63.5 (OCH_2), 65.0 (pyrazole C-4), 119.1 (NPh C-2,6), 125.6 (NPh C-4), 128.2 (CPh C-3,5), 128.3 (CPh C-2,6), 128.4 (NPh C-3,5), 130.20 (CPh C-1), 130.22 (CPh C-4), 136.8 (NPh C-1), 153.0 (pyrazole C-3), 162.1 (ester CO), 165.6 (pyrazole C-5). ^{15}N NMR (40 MHz, $CDCl_3$): δ (ppm) −189.9 (N-1), −53.7 (N-2). MS: *m/z* (%) = 614 $[M]^+$ (12), 307 (61), 263 (23), 262 (100), 233 (79), 129 (31), 91 (41), 77 (100). HRMS (ESI): calcd. for $C_{36}H_{30}N_4O_6Na^+$ $[M+Na]^+$ 637.2058; found 637.2057.

5-Hydroxy-1-phenyl-1*H*-pyrazole-4-carbohydrazide (**5**).

A suspension of ethyl 5-hydroxy-1-phenyl-1*H*-pyrazole-4-carboxylate (**1a**) (2.32 g, 10 mmol) in 5 mL of 85% hydrazine hydrate was heated at 105° for 4 h. After evaporation of the solvents under reduced pressure a yellowish, viscous oil was obtained which was purified by column chromatography (SiO_2, CH_2Cl_2). Yield: 1.40 g (64%), yellow crystals, mp 38–40 °C. ^1H NMR (400 MHz, $CDCl_3$): δ (ppm) 4.86 (br s together with H_2O, NH_2), 6.97 (m, 1H, Ph H-4), 7.27 (m, 2H, Ph H-3,5), 7.44 (s, 1H, pyrazole H-3), 8.08 (m, 2H, Ph H-2,6), 9.16 (s, 1H, NH). ^{13}C NMR (100 MHz, $CDCl_3$): δ (ppm) 93.9 (C-4), 117.7 (Ph C-2,6), 122.1 (Ph C-4), 128.2 (Ph C-3,5), 139.4 (pyrazole C-3), 141.6 (Ph C-1), 164.0 (pyrazole C-5), 166.1 (CO). ^{15}N NMR (40 MHz, $CDCl_3$): δ (ppm) −332.0 (NH_2), −264.8 (NH), −184.4 (N-1), −98.4 (N-2). HRMS (ESI): calcd. for $C_{10}H_{10}N_4O_2Na^+$ $[M+Na]^+$ 241.0696; found 241.0695.

4-Chloro-4-(dichloromethyl)-5-methyl-2-phenyl-2,4-dihydro-3*H*-pyrazol-3-one (**7**).

The corresponding 5-hydroxy-3-methyl-1-phenyl-1*H*-pyrazole-4-carbaldehyde (**6**) (2.02 g, 10.0 mmol) [8] and $SOCl_2$ (7.25 mL, 11.9 g, 100 mmol) were heated to reflux for 3 h. Then the excess $SOCl_2$ was removed under reduced pressure, toluene (ca. 10 mL) was added, and the solvent was removed under reduced pressure. The remaining residue was washed with *n*-hexane and dried *in vacuo*. Yield: 0.68 g (23%) yellow liquid. ^{1}H NMR (400 MHz, $CDCl_3$): δ (ppm) 2.43 (s, 1H, 5-Me), 5.99 (s, 1H, $CHCl_2$), 7.25 (m, 1H, Ph H-4), 7.43 (m, 2H, Ph H-3,5), 7.86 (m, 2H, Ph H-2,6). ^{13}C NMR (100 MHz, $CDCl_3$): δ (ppm) 15.1 (5-Me), 66.5 (C-4), 70.0 ($CHCl_2$), 119.0 (Ph C-2,6), 126.1 (Ph C-4), 129.1 (Ph C-3,5), 136.9 (Ph-1), 154.8 (C-5), 165.9 (C-3). ^{15}N NMR (40 MHz, $CDCl_3$): δ (ppm) −197.6 (N-2), −55.9 (N-1). MS: *m/z* (%) = 290/292/294/296 $[M]^+$ (23/22/6/1), 207 (57), 77 (100), 51 (33). HRMS (ESI): calcd. for $C_{11}H_9Cl_3N_2ONa^+$ $[M+Na]^+$ 312.9673; found: 312.9665.

Reaction of **1a** with sulfuryl chloride

Ethyl 5-hydroxy-1-phenyl-1*H*-pyrazole-4-carboxylate (**1a**) (0.65 g, 2.8 mmol) was added to sulfuryl chloride (4.04 mL, 50 mmol) and the solution was refluxed for 3 h. The reaction mixture was cooled and 10 mL of toluene was added. The solvent was evaporated under reduced pressure and the residue was subjected to flash chromatography (CH_2Cl_2/light petroleum = 1:1, v/v).

Ethyl 4-chloro-1-(4-chlorophenyl)-5-oxo-4,5-dihydro-1*H*-pyrazole-4-carboxylate (**8**)

Yield: 228 mg (27%), yellowish crystals, mp 42–43 °C. ^{1}H NMR (400 MHz, $CDCl_3$): δ (ppm) 1.32 (t, ^3J = 7.1 Hz, 3H, CH_3), 4.35 (q, ^3J = 7.1 Hz, 2H, OCH_2), 7.40 (m, 2H, Ph H-3,5), 7.51 (s, 1H, H-3), 7.82 (m, 2H, Ph H-2,6). ^{13}C NMR (100 MHz, $CDCl_3$): δ (ppm) 13.9 (CH_3), 63.3 (pyrazole C-4), 64.7 (OCH_2), 120.1 (Ph C-2,6), 129.2 (Ph C-3,5), 131.6 (Ph C-4), 135.5 (Ph C-1), 145.8 (pyrazole C-3), 161.1 (ester CO), 164.4 (pyrazole C-5). ^{15}N NMR (40 MHz, $CDCl_3$): δ (ppm) −197.5 (N-1), −44.3 (N-2). MS: *m/z* (%) = 300/302/304 $[M]^+$ (5/3/0.5), 198 (32), 196 (32), 139 (26), 113 (21), 111 (65), 107 (28), 97 (34), 85 (28), 83 (24), 79 (44), 77 (33), 75 (40), 71 (54), 69 (41), 67 (25), 63 (26), 62 (23), 57 (100), 56 (25), 55 (64), 53 (33), 51 (24), 50 (23), 43 (76), 41 (50). HRMS (ESI): calcd. for $C_{12}H_{10}Cl_2N_2O_3Na^+$ $[M+Na]^+$ 322.9961; found 322.9969.

4,4-Dichloro-2-(4-chlorophenyl)-2,4-dihydro-3*H*-pyrazol-3-one (**9**)

Yield: 266 mg (36%), light red crystals, mp 38–40 °C.^{1}H NMR (400 MHz, $CDCl_3$): δ (ppm) 7.41 (m, 2H, Ph H-3,5), 7.63 (s, 1H, pyrazole H-3), 7.80 (m, 2H, Ph H-2,6). ^{13}C NMR (100 MHz, $CDCl_3$): δ (ppm) 70.7 (pyrazole C-4), 120.0 (Ph C-2,6), 129.3 (Ph C-3,5), 131.8 (Ph C-4), 135.1 (Ph C-1), 146.8 (pyrazole C-3), 163.2 (pyrazole C-5). ^{15}N NMR (40 MHz, $CDCl_3$): δ (ppm) −203.5 (N-1), −54.9 (N-2). MS: *m/z* (%) = 262/264/266/268 $[M]^+$ (32/31/10/1), 229 (29), 227 (44), 200 (22), 198 (70), 196 (72), 139 (41), 132 (26), 113 (33), 111 (100), 97 (37), 79 (38), 77 (30), 75 (48), 63 (31), 62 (32), 61 (21), 60 (21), 51 (27), 50 (31), 43 (24).

General procedure for the preparation of bipyrazoles 10

A suspension of diester **3** (2.0 mmol), EtOH (5 mL), and 35% aq KOH (5 mL) was refluxed for 3 h. The deep colored solution was concentrated under reduced pressure and acidified with

conc. HCl. Then the solution was cooled in the refrigerator for 30 min, the formed precipitate was filtered off, washed with water (3 × 10 mL) and dried.

1,1'-Diphenyl-1*H*,1'*H*-[4,4'-bipyrazole]-5,5'-diol (**10a**) [9]

Yield: 0.59 g (93%), reddish crystals; mp 191–202 °C. ^{1}H NMR (300 MHz, DMSO-d_6): δ (ppm) 3.73 (br s, OH, together with trace H_2O), 7.31 (m, 2H, Ph H-4), 7.50 (m, 4H, Ph H-3,5), 7.75 (m, 4H, Ph H-2,6), 7.96 (s, 2H, pyrazole H-3). ^{13}C NMR (75 MHz, DMSO-d_6): δ (ppm) 97.5* (C-4), 120.8 (Ph C-2,6), 126.2 (Ph C-4), 129.1 (Ph C-3,5), 136.9 (Ph C-1), 133.7* (C-3), 155.4* (C-5); *broad or very broad signal. IR (KBr): 3406 cm^{-1}. MS: *m/z* (%) = 318 $[M]^+$ (52), 93 (25), 77 (100). Anal. Calcd for $C_{18}H_{14}N_4O_2$·0.4 H_2O: C, 66.41; H, 4.58; N, 17.21. Found: C, 66.14; H, 4.38; N, 16.98. HRMS (ESI): calcd. for $C_{18}H_{15}N_4O_2^+$ $[M+H]^+$ 319.1190; found 319.1191.

1,1'-Bis(4-bromophenyl)-1*H*,1'*H*-[4,4'-bipyrazole]-5,5'-diol (**10c**)

Yield: 0.62 g (65%), reddish crystals; mp 270–290 °C. ^{1}H NMR (400 MHz, DMSO-d_6): δ (ppm) 7.68 (m, 4H, Ph H-3,5), 7.73 (m, 4H, Ph H-2,6), 7.97 (s, 2H, pyrazole H-3). ^{13}C NMR (100 MHz, DMSO-d_6): δ (ppm) 97.3 (C-4), 118.4 (Ph C-4), 122.3 (Ph C-2,6), 132.0 (Ph C-3,5), 134.4 (C-3, ^{1}J = 187.5 Hz), 136.3 (Ph C-1), 155.6 (C-5). ^{15}N NMR (40 MHz, DMSO-d_6): δ (ppm) −196.2 (N-1); N-2 was not found. ESI-MS: *m/z* (%) = 477 $[M+H]^+$ (100), 475 (47). HRMS (ESI): calcd. for $C_{18}H_{13}Br_2N_4O_2^+$ $[M+H]^+$ 474.9400; found 474.9401.

1,1'-Bis(3,4-dichlorophenyl)-1*H*,1'*H*-[4,4'-bipyrazole]-5,5'-diol (**10d**)

Yield: 0.84 g (92%); reddish crystals; mp 228–230 °C. ^{1}H NMR (300 MHz, DMSO-d_6): δ (ppm) 5.70 (br s, 2H, OH), 7.70 (d, ^{3}J = 8.9 Hz, 2H, Ph H-5), 7.80 (dd, ^{3}J = 8.9 Hz, ^{4}J = 2.4 Hz, 2H, Ph H-6), 7.99 (s, 2H, pyrazole H-3), 8.04 (d, ^{4}J = 2.4 Hz, 2H, Ph H-2). ^{13}C NMR (75 MHz, DMSO-d_6): δ (ppm) 97.4 (C-4), 119.7 (Ph C-6), 121.2 (Ph C-2), 127.8 (Ph C-4), 130.9 (Ph C-5), 131.4 (Ph C-3), 135.0 (C-3, ^{1}J = 188.0 Hz), 136.7 (Ph C1), 155.7 (C-5). ^{15}N NMR (50 MHz, DMSO-d_6): δ (ppm) −197.8 (N-1); N-2 was not found. IR (KBr): 3425 (OH) cm^{-1}. MS: *m/z* (%) = 459 (45), 457 $[M+H]^+$ (100), 455 (75), 255 (42). HRMS (ESI): calcd. for $C_{18}H_{11}Cl_4N_4O_2^+$ $[M+H]^+$ 454.9631; found 454.9626.

3,3'-Dimethyl-1,1'-diphenyl-1*H*,1'*H*-[4,4'-bipyrazole]-5,5'-diol (**10h**)

Yield: 0.60 g, 87%; reddish crystals; mp 290–330 °C. (lit. [10] mp > 300 °C). ^{1}H NMR (300 MHz, DMSO-$d_{6,}$): δ (ppm) 2.16 (s, 6H, 3-Me), 7.21 (m, 2H, Ph H-4), 7.44 (m, 4H, Ph H-3,5), 7.78 (m, 4H, Ph H-2,6), 11.40 (br s, 2H, OH). ^{13}C NMR (75 MHz, DMSO-d_6): δ (ppm) 13.3* (3-Me), 95.0* (C-4), 119.9* (Ph C-2,6), 124.9* (Ph C-4), 128.8* (Ph C-3,5), 138.1* (Ph C-1), 146.8* (C-3); C-5 was not found; *broad or very broad signal. IR (KBr): 3426 (OH) cm^{-1}. MS (ESI): *m/z* (%) = 348 (26), 347 $[M+H]^+$ (100). Anal. Calcd for $C_{20}H_{18}N_4O_2$·0.5 H_2O: C, 67.59; H, 5.39; N, 15.76; found: C, 67.78; H, 5.18; N, 15.79. HRMS (ESI): calcd. for $C_{20}H_{19}N_4O_2^+$ $[M+H]^+$ 347.1503; found 347.1504.

X-ray analysis of compound **3a**

The X-ray intensity data was measured on a Bruker-Nonius KappaCCD diffractometer equipped with graphite monochromator, Mo K/α INCOATEC micro focus sealed tube and Cryostream 700 cooling device. The structure was solved by *direct method* and refined by *full-matrix least-squares techniques*. Non-hydrogen atoms were refined with *anisotropic displacement parameters*. Hydrogen atoms were fitted to the peaks of the difference synthesis as well as calculated geometrically and refined with a riding model. The following software was used: *DENZO and HKL SCALEPEAK software package* [11] using a narrow-frame algorithm for frame integration, no absorption correction due to small absorption coefficient, *SIR-92* [12] for structure solution, refinement molecular diagrams and graphical user-interface, *maXus* [13] for refinement and graphical user-interface, *Platon* [14] for symmetry check and Cg(Pi-Ring) interactions calculations. Experimental data and CCDC-code can be found in Table S1. Crystal data, data collection parameters, and structure refinement details are given in Tables S2 and S3. Molecular structure in "Ortep View" is displayed in Figure S1 and arrangement of enantiomers in the crystal is displayed in Figure S2.

Table S1: Experimental parameter and CCDC-Code.

Sample	Machine	Source	Temp.	Detector Distance	Time/ Frame	#Frames	Frame width	CCDC
			[K]	[mm]	[s]		[°]	
**3a**	Bruker-Nonius KappaCCD	Mo	153	32	30	325	1.2	979625

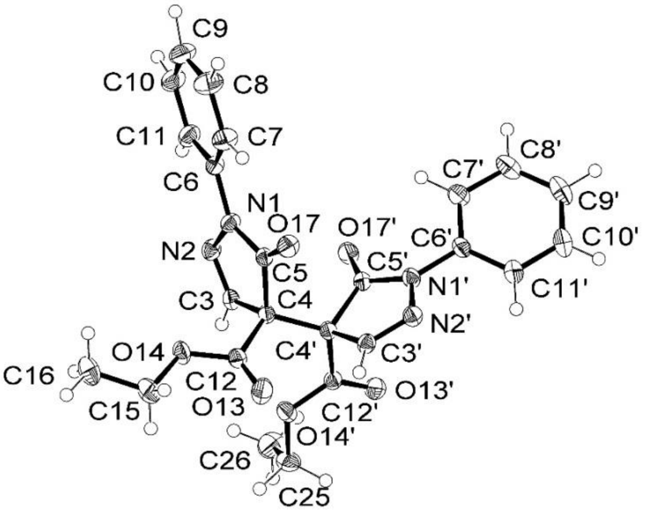

Figure S1: Asymmetric unit of compound **3a** (4*S*,4'*S* enantiomer), drawn with 50% displacement ellipsoids.

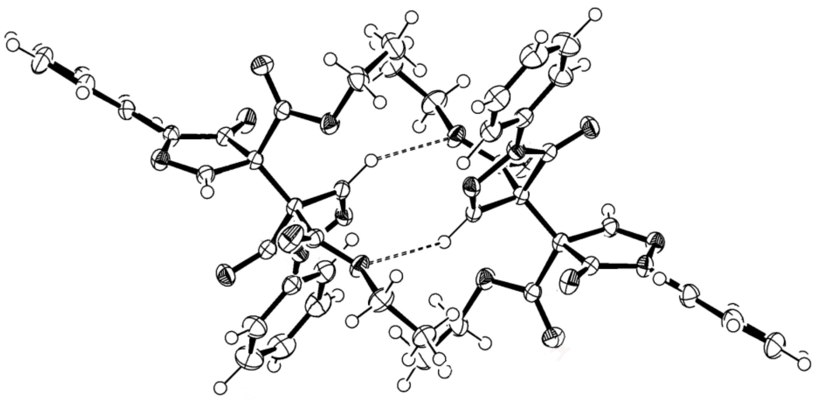

Figure S2: Arrangement of enantiomers 4*R*,4'*R* and 4*S*,4'*S* in the crystal of **3a**, drawn with 50% displacement ellipsoids.

Table S2: Sample and crystal data of compound **3a**.

**Chemical formula**	$C_{24}H_{22}N_4O_6$	**Crystal system**	triclinic	
**Formula weight [g/mol]**	462.46	**Space group**	P 1	
**Temperature [K]**	153	**Z**	2	
**Measurement method**	\Φ and \ω scans	**Volume [Å^3]**	1107.45(4)	
**Radiation (Wavelength [Å])**	MoKα (λ = 0.71073)	**Unit cell dimensions [Å] and [°]**	10.6975(2)	68.7348(8)
**Crystal size / [mm^3]**	0.48 × 0.31 × 0.28		10.7160(2)	68.1795(7)
**Crystal habit**	Colourless prism		11.4884(2)	70.2362(8)
**Density (calculated) / [g/cm^3]**	1.387	**Absorption coefficient / [mm^{-1}]**	0.1	
**Abs. correction Tmin**	-	**Abs. correction Tmax**	-	
**Abs. correction type**	no absorption correction	**F(000) [e$^-$]**	484	

Table 3. Data collection and structure refinement of compound **3a**.

**Index ranges**	-13 ≤ h ≤ 14, -13 ≤ k ≤ 14, -12 ≤ l ≤ 15	**Theta range for data collection [°]**	2.0 to 28.0	
**Reflections number**	5319	**Data / restraints / parameters**	4245/0/307	
**Refinement method**	Least squares matrix: full	**Final R indices**	all data	R1 = 0.068, wR2 = 0.294
**Function minimized**	Σ w(F$_o^2$ - F$_c^2$)^2		I>3σ(I)	R1 = 0.047, wR2 = 0.193
**Goodness-of-fit on F^2**	1.028	**Weighting scheme**	w=1/(s^2(F_o^2)+0.10000F_o^2)	
**Largest diff. peak and hole [e Å^{-3}]**	0.48/-0.53			

References

1. Stanovnik, B.; Tisler, M.; Bradac, J.; Budic, B.; Koren, B.; Mozetic-Rescic, B. *Heterocycles* **1979**, *12*, 457-460. doi:10.3987/R-1979-04-0457
2. Begtrup, M. *Acta Chem. Scand.* **1974**, *28B*, 61-77. doi:10.3891/acta.chem.scand.28b-0061
3. Taylor, A. W.; Cook, R. T. *Tetrahedron* **1987**, *43*, 607-616. doi:10.1016/S0040-4020(01)89994-4
4. Spassova, M. K.; Zakharieva, R. D. *Collect. Czech. Chem. Commun.* **1989**, *54*, 196-199. doi:10.1135/cccc19890196
5. Holzer, W.; Krca, I. *Heterocycles* **2003**, *60*, 2323-2342. doi:10.3987/COM-03-9857
6. Pavlik, J. W.; Ervithayasuporn, V.; MacDonald, J. C.; Tantayanon, S. *ARKIVOC*, **2009**, (viii), 57-68.
7. Chande, M. S.; Bhat, U. S. *Indian J. Chem., Sect. B: Org. Chem. Incl. Med. Chem.* **2006**, *45B*, 1041-1047.
8. Kurkovskaya, L. N.; Shapet'ko, N. N.; Samartseva, E. D.; Koshelev, Yu. N.; Sof'ina, E. M.; Kvitko, I. Ya. *Zh. Org. Khim*. **1975**, *11*, 1734-1739.
9. Obara, H.; Igarashi, T.; Sakurai, K.; Oi, T. U.S. US 6121305 A (Sep 19, 2000).
10. Azev, Y. A.; Gryazeva, O. V.; Golomolzin, B. V. *Chem. Heterocycl. Compd. (N. Y., NY, U. S.)* **2003**, *39*, 1478-1486. doi:10.1023/B:COHC.0000014412.48911.60
11. Otwinowski, Z.; Minor, W. Processing of X-ray diffraction data collected in oscillation mode. In *Methods in Enzymology;* Carter, C. W. Jr.; Sweet, R. M., Eds.; Academic Press: New York, 1997; Vol. 276, pp. 307–326. doi:10.1016/S0076-6879(97)76066-X
12. Altomare, A.; Cascarano, G.; Giacovazzo, C.; Guagliardi, A.; Burla, M. C.; Polidori, G.; Camalli, M. *J. Appl. Crystallogr.* **1994**, *27*, 435–436. doi:10.1107/S0021889894000221
13. Mackay, S.; Gilmore, C. J.; Edwards, C.; Stewart, N.; Shankland, K. *maXus Computer Program for the Solution and Refinement of Crystal Structures*. Bruker Nonius, The Netherlands, MacScience, Japan & The University of Glasgow, 1999.
14. Spek, A. L. *Acta Crystallogr., Sect. D: Biol. Crystallogr.* **2009**, *65*, 148–155. doi:10.1107/S090744490804362X
